# Phase-noise and stability comparison of fiber-loop and integrated-waveguide coupling in optomechanical crystal oscillators

**DOI:** 10.1038/s41598-026-55778-1

**Published:** 2026-06-04

**Authors:** Raúl Ortiz, Xinyan Yang, Alejandro Martínez, Laura Mercadé

**Affiliations:** Nanophotonics Technology Center, Universitat Politècnica de València, Valencia, 46022 Spain

**Keywords:** Optics and photonics, Physics

## Abstract

Optomechanical crystal cavities (OMCCs) display appealing features to function as on-chip photonic microwave oscillators (PMOs) when operated in the phonon lasing regime. Silicon OMCCs have so far demonstrated the best performance in terms of phase noise in a free-running configuration. Some preliminary experiments have also demonstrated that the generated microwave tone can be used for mixing wireless signals compliant with 5G standards. Still, more research is needed to identify the primary noise sources and determine the path towards improvement. Here, we report experiments on the realization of optomechanical PMOs operating at 4 GHz under two different driving conditions of the cavity: light coupling via a tapered fiber loop versus using an adjacent integrated waveguide illuminated by a lensed fiber. We performed measurements of the phase noise and the frequency stability in time of the detected tone for both cases and observed an improvement when the light is coupled by an adjacent integrated waveguide. This can be explained by the fact that the ambient-induced motion and changes due to perturbations in the refractive index of the medium surrounding the fiber loop result in variations of the coupling, which in turn affect the stability of the PMO. These results emphasize the relevance of a mechanically stable coupling technique when using such optomechanical devices in real applications, which can be of particular importance in environments where large vibrations are expected.

## Introduction

Photonic microwave oscillators (PMOs) are optoelectronic devices that enable the generation of highly-stable microwave tones whilst doing the processing entirely in the optical domain. PMOs display multiple advantages over their electronic counterparts, such as immunity to electromagnetic interference, compactness, low weight, long-distance transport using optical fiber links, and extremely low noise^1^. While the first demonstrations used long paths of optical fiber to produce self-sustained oscillations at the desired microwave frequency, the last few years have witnessed a trend towards miniaturization by implementing PMOs in photonic integrated circuits (PICs).

Amongst the different approaches to implement integrated PMOs, soliton microcombs resulting from exploiting nonlinear effects and tailored dispersion in ring resonators show an exquisite behavior in terms of stability and frequency noise, both as a free-running oscillator^2^ or as a part of a more complex closed-loop technique known as optical frequency division^3–5^. However, achieving microwave frequencies below 15 GHz requires both very large footprints (over 1 mm ring radii) and large (above 100 mW) driving powers. For example, the microcombs in^6^ require an optical power of 160 mW and ring lengths of about 1.5 cm to generate a microwave tone of 10.6 GHz upon photodetection. This can severely limit the use of microcomb-based PMOs in applications requiring extreme compactness, low weight, low power consumption, and operation below 15 GHz (as in terrestrial and satellite wireless networks and systems).

Reaching microwave frequencies below 15 GHz is much easier by using optomechanical crystal cavities (OMCCs)^7^. Such cavities simultaneously confine light and mechanical waves in wavelength-scale dielectric regions, enabling high coupling between both fields under proper design^8^. When operating in the blue-detuned regime, such cavities can be driven to the so-called phonon lasing (or self-sustained oscillations) regime, where a very stable microwave tone at the frequency of the mechanical resonance of the cavity (usually, in the range of several GHz) modulates the input laser and can produce a low noise microwave tone after photodetection^9^. Although the phase noise is not as small as in the case of microcombs, the driving power is much lower, typically below 1 mW. For instance, considering the case of a free-running configuration, an InGaP OMCC operating at *≈* 3 GHz was demonstrated to operate under very low input powers (< 0.1 mW)^10^; and a silicon OMCC operating at 4 GHz showed a phase noise below −100 dBc/Hz at 100 kHz^9^. As in microcombs, locking to an external source in a closed-loop configuration can dramatically enhance the stability (30 dB reduction of the phase noise in a GaP OM cavity^11)^, with the advantage over microcombs that it can be done via electro-mechanical self-injection (simpler and less expensive than using optical sources as in optical frequency division). Furthermore, when driven by external signals, optomechanical oscillators have been shown to exhibit robust synchronization and entrainment even at high-order harmonics, enabling functionalities such as purely optomechanical frequency division^12^. More advanced control strategies, such as Floquet engineering, enable dynamically tunable and multistable synchronization, providing access to complex phase dynamics and reconfigurable oscillator networks^13^.

The free-running configuration of PMOs is, in general, less complex (in terms of hardware) and has a smaller footprint and a lower weight than its closed-loop counterpart. To the best of our knowledge, the lowest phase noise value in free-running OMCCs reported to date was measured in the full-phononic bandgap OMCC demonstrated in^9^. Using this cavity, several experiments on frequency mixing of data signals in the optical domain using the microwave tone as a local oscillator have been reported^14,15^. Remarkably, mixing of 5G data signals has been demonstrated to be feasible, but the phase noise limits the number of bits per symbol to 4 (16-QAM modulation) in the modulation of the subcarriers of the used OFDM data signal. In all those experiments, the OMCC was driven using the evanescent field of a tapered fiber loop placed in proximity to the cavity. This approach, useful for lab measurements, may result in a reduction of the microwave tone stability, since tiny alterations of the environment may slightly displace the fiber and, therefore, change the excitation conditions. Similar behavior has been reported in other optomechanical systems employing tapered fiber coupling^16^, where phase instability in phonon lasing of a microtoroid resonator was attributed to mechanical fluctuations associated with the tapered fiber motion. This could be solved by exciting the OMCC via a parallel integrated waveguide, which would be mechanically more stable than the fiber loop and would also pave the way towards system packaging. However, no previous experiments on the phase noise of the silicon OMCC presented in^9^ but excited via an integrated waveguide have been reported.

Here, we characterize the performance of this silicon OMCC as a PMO using the two mentioned coupling approaches: tapered fiber-loop and integrated waveguide excited via a lensed fiber. We show that the cavity can be driven to its phonon lasing regime in both configurations and measure the phase noise, the Allan deviation, and the time jitter of both cavities. Our results confirm that measurements performed via the integrated waveguide coupled to the lensed fiber are more stable and exhibit less drift in both the phase noise and Allan deviation characterizations.

## Results

### OMCC structure

**Fig. 1:**
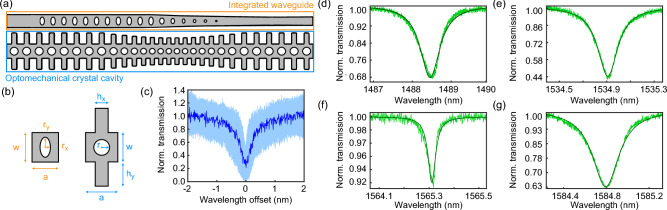
Optical and mechanical response of the OMCC under study. (**a**) Schematic of the silicon OMCC showing the mirror cells, transition cells, and defect region. (**b**) Geometry of a unit cell. The thickness of the cavity is 220 nm. For the defect region, the parameters are *w = 570 *nm, *a = 325 *nm, $h_x = 163\,$nm, $h_y = 250\,$nm, and *r = 98 *nm. For the mirror cells, the values are *w = 570 *nm, *a = 500 *nm, $h_x = 200\,$nm, $h_y = 450\,$nm, and *r = 150 *nm. (**c**) Mean and standard deviation of the optical resonance corresponding to $\lambda \approx 1488\;$nm measured with the loop setup. (**d**-**g**) Normalized optical transmission spectra for different cavity resonances, fitted with Lorentzian profiles. The corresponding quality factors are: (**d**) $Q=2.42\times 10^3$, (**e**) $Q=7.77\times 10^3$, (**f**) $Q=4.06\times 10^4$, (**g**) $Q=5.73\times 10^3$.

The OMCC is designed to confine both optical and mechanical modes at its defect region, where both kinds of waves can interact. The cavity is engineered to exhibit a TE-photonic bandgap in the mirror regions around 1550 nm, and a full phononic bandgap centered around 3.5 GHz. This dual-bandgap design enables the simultaneous localization of optical and mechanical modes, which is essential to achieve strong optomechanical coupling and to operate in the phonon lasing regime under appropriate optical driving. The cavity is patterned with a periodic arrangement of unit cells, each consisting of two lateral corrugations and a central circular hole. To create a defect region, the unit cell parameters are adiabatically varied from the mirror regions at the cavity edges toward the defect region in the center. This transition ensures efficient mode confinement while minimizing scattering losses. A schematic of the OMCC geometry, showing the mirror cells, transition cells, and defect region, is displayed in Fig. 1(a), with a close view of the unit cell parameters depicted in Fig. 1(b). More details about the optical and mechanical design of this OMCC are reported in^9^. The defect region can usually support multiple optical resonances with high quality factors. Figure 1(c) displays the results of four independent measurements of the same optical resonance at $\lambda \approx 1488\;$nm using the fiber-loop setup, highlighting noticeable variations between the datasets due to different relative positions between the fiber loop and the cavity, thus changing the refractive index of the medium surrounding the system. Additionally, the OMCC investigated in this work exhibits three further optical resonances within the wavelength range explored in the experiments, as illustrated in Figs. 1(d)–(g). These optical resonances were measured via the edge setup, resulting in stable measurements with reduced drift, as there is no change in the medium surrounding the cavity. It is worth noting that all measurements in both setups were performed on the same cavities within the same device. The loaded Q factors are listed in the figure caption.

**Fig. 2:**
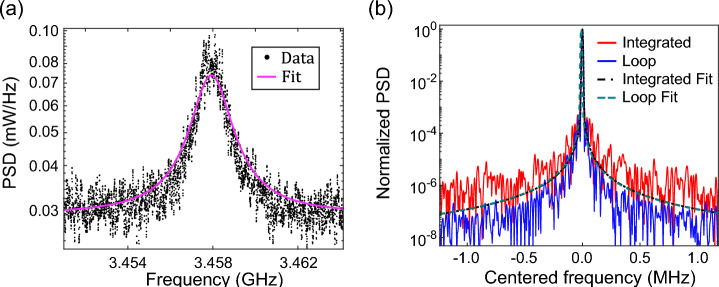
Transduction of the mechanical resonance. (**a**) Low-power transduced mechanical mode, fitted with a Lorentzian curve. The mechanical quality factor is $Q_m=1647$. (**b**) Normalized power spectral density (PSD) of the mechanical mode for the fiber-loop and edge coupling configurations, fitted with Voigt profiles. The measured resonance frequencies are $\Omega /2\pi = 3.48$ GHz (fiber-loop) and $\Omega /2\pi = 3.50$ GHz (integrated waveguide). This small discrepancy arises because the optical power coupled into the cavity is not exactly the same in both configurations. Due to optomechanical backaction, slight variations in the injected power lead to small shifts of the mechanical frequency, which are therefore expected.

The OMCC also has a mechanical mode at GHz frequencies, which can be transduced when driving the cavity with a laser tone having a wavelength close to the optical resonance, Fig. 2(a). Figure 2(b) presents a comparison of the normalized power spectral density (PSD) of the GHz mechanical mode at $\Omega \approx 3.5$ GHz obtained after photodetection for both coupling configurations described in the following section. The measurements in Fig. 2(b) were performed in the phonon lasing regime, which is reached by increasing the input optical power and properly tuning the laser detuning. In this regime, optomechanical dynamical backaction leads to a reduction of the effective mechanical damping, which can become negative, resulting in self-sustained coherent oscillations of the mechanical mode. This gives rise to a pronounced narrowing of the mechanical linewidth and the appearance of nonlinear features in the spectrum, such as harmonic generation, characteristic of this regime^17–19^. Noticeably, both setups exhibit similar behavior in the transduction of the mechanical resonance. The fits indicate first that the mechanical motion can be efficiently transduced and second that linewidths of the resonances remain comparable, confirming the robustness of the mechanical mode under different coupling conditions.

### Sample fabrication

**Fig. 3:**
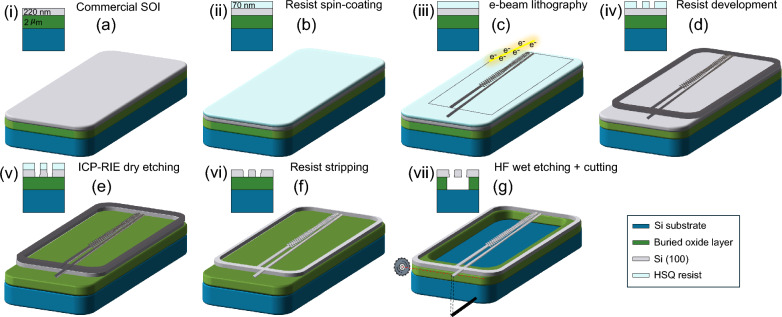
Fabrication process of edge-coupled OMCCs with negative resist. (**a**) Starting SOI wafer with *220 *nm monocrystalline silicon and $2\;\mu$m buried oxide. (**b**) Spin-coating and baking of *70 *nm HSQ negative resist. (**c**) Pattern definition by electron beam lithography. (**d**) Development of exposed resist in a mix of Methyl Isobutyl Ketone and Isopropyl Alcohol. (**e**) Pattern transfer into silicon by reactive ion etching (ICP-RIE) using $SF_6/C_4F_8$ gases. (**f**) Resist stripping of the PMMA layer on the cavity. (g) Removal of the buried oxide layer by wet etching in buffered hydrofluoric acid to release the suspended nanobeams and the cutting process. (i)-(vii) Cross-sectional views of (**a-g**) steps.

OMCCs were fabricated on a silicon-on-insulator (SOI) wafer using fabrication techniques well-described elsewhere^20^. Specifically, we started with a SOI wafer, comprising a *220 *nm thick monocrystalline silicon layer over a $2\;\mu$m buried oxide layer, supported on a bulk silicon substrate (Fig. 3(a)). To fabricate the devices, a negative resist (HSQ) was spin-coated and patterned by electron beam lithography to define the cavities as well as their access waveguides extending to the chip edge, (Fig. 3(b)). The desired pattern was then defined by electron beam lithography, where the resist is locally exposed to a focused electron beam performed with a Raith tool (e-beam Raith 150) (Fig. 3(c)). After exposure, the resist was developed, revealing the underlying silicon in the non-patterned areas, Fig. 3(d). The patterned resist layer acts as a mask during the subsequent etching of the silicon device layer. This step is performed by reactive ion etching (ICP-RIE) with a gas mixture of $SF_6/C_4F_8$, which transfers the pattern into the silicon and defines the suspended nanobeams (Fig. 3(e)). Then, a resist stripping was performed to remove the resist layer on the beam (Fig. 3(f)). The buried oxide layer beneath the nanobeams was removed by a wet etching step in buffered hydrofluoric (HF) acid, releasing the silicon structures from the substrate to and achieving the nanobeam suspension that enables mechanical motion^21^, as seen in Fig. 3(g). Since the employed electron-beam system cannot expose patterns directly at the chip edge, the wafer was finally diced at the very end of the flow, requiring the lithography to be completed first and the facet to be opened afterwards by dicing to obtain a clean and reproducible termination.

**Fig. 4:**
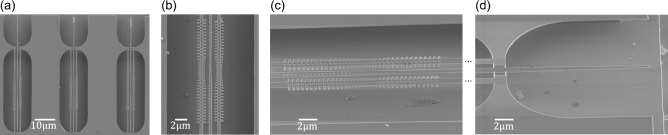
Fabricated OMCCs. (**a**) SEM image of three OMCCs coupled to the same bus waveguide. (**b**) Close-up of the OMCCs and the tapered Bragg mirror. (**c**) Tilted view of the interaction region. (**d**) Tilted view of the chip facet with the bus waveguide.

Figure 4(a)–(d) present different scanning electron microscope (SEM) images of the fabricated sample containing a set of OMCC pairs coupled to an input waveguide. The two OMCCs coupled to a single waveguide were slightly scaled to separate the optical and mechanical resonances of each cavity spectrally. The reason is that this way we should be able to observe optical and mechanical resonances in two cavities during a single measurement procedure, therefore optimizing time and resources. The silicon waveguide used to excite the OMCCs is ended in a tapered Bragg mirror on one side to ensure efficient coupling to the waveguide^22^ and in an inverted taper on the other side to achieve efficient coupling from an external fiber in the case of waveguide coupling. In particular, Fig. 4(a) shows three pairs of OMCCs coupled to three bus waveguides. A magnified SEM view of one of the OMCC pairs is shown in Fig. 4(b), highlighting the tapered Bragg mirror that reflects the light and enables lateral coupling into the cavities. Figure 4(c) and (d) show tilted SEM views of the coupling region and the waveguide facet, respectively, illustrating the precise alignment and fabrication quality of the structures.

### Experimental setups

**Fig. 5:**
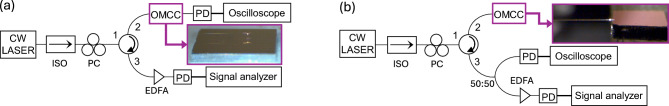
Description of the experimental setups. (**a**) Fiber-loop coupling scheme: a tapered fiber loop couples evanescently to the OMCC. Inset: microscope image of the fiber loop aligned to the cavity. (**b**) Integrated waveguide coupling scheme: a lensed fiber couples light into the bus silicon waveguide, which excites the OMCC via evanescent coupling aided by a Bragg mirror. Inset: microscope image of the fiber-to-chip coupling.

Figure 5(a) and (b) sketch the two experimental configurations used to characterize the OMCCs: fiber-loop coupling and integrated waveguide coupling. In the fiber-loop configuration (Fig. 5(a)), a tapered optical fiber is first fabricated to efficiently couple light into the cavity. To this end, a *∼*3 cm region of standard SMF-28 optical fiber is stripped from its protective coating using a fiber stripper and clamped at both ends. The exposed section is then heated with a hydrogen flame while being pulled at constant speed by motorized stages. As the fiber is stretched under heating, its diameter decreases, leading to a transition from multimode guidance to a single-mode tapered fiber. A loop is subsequently formed at the center of the taper, allowing evanescent coupling to the OMCC. During measurements, the fiber loop is carefully aligned to the cavity and brought into gentle contact with the sample at the central region of the cavity, as shown in the inset of Fig. 5(a). Importantly, contact with the lateral corrugations of the OMCC is avoided to preserve their mechanical freedom of the nanobeam and ensure its motion at the corresponding vibrational frequency. In this setup, light from a continuous-wave (CW) laser passes through an optical isolator (ISO) and a polarization controller (PC) before entering port 1 of an optical circulator. At port 2, light couples to the OMCC via the fiber loop. The transmitted optical signal is photodetected and monitored with an oscilloscope, while the reflected signal is routed to port 3 of the circulator, amplified with an erbium-doped fiber amplifier (EDFA), and then photodetected. This amplified signal is subsequently analyzed with a radio-frequency signal analyzer (RSA) to measure the mechanical response. Interestingly, this scheme enables simultaneous monitoring of both the optical transmission and the mechanically-induced microwave signal.

The waveguide-coupling setup (Fig. 5(b)) relies on butt-coupling to an inverted taper from a lensed fiber. The CW laser output first passes through an ISO and then into a PC before entering port 1 of the circulator. At port 2, the light is directed towards the chip and injected into the bus waveguide via a lensed fiber aligned to its input facet (inset in Fig. 5(b)). Light is reflected at the Bragg mirror at the end of the bus waveguide and laterally coupled into the OMCC. The signal collected back into the lensed fiber is routed to port 3 of the circulator and subsequently divided by a 50:50 coupler. One output is directed to a photodiode and monitored with an oscilloscope for the optical response, while the other is amplified with an EDFA, photodetected, and analyzed with the RSA to measure the mechanical response. The EDFA, therefore, compensates for the weak signal returning from the chip, ensuring sufficient sensitivity in the detection of the mechanical spectrum.

## Discussion

**Fig. 6:**
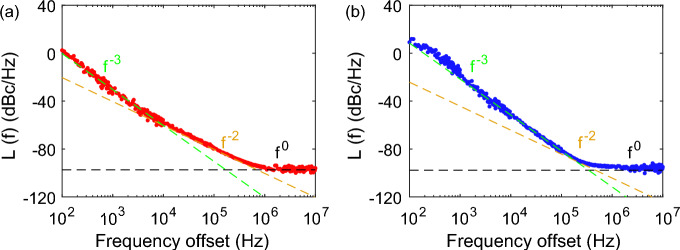
Phase noise spectra of the measured OMCC and characteristic slopes according to Leeson’s model. (**a**) Edge-coupled OMCC. (**b**) Loop-coupled OMCC.

In this section, we compare the frequency stability of the microwave tone obtained with the fiber-loop and waveguide-coupling (or edge setup) configurations. To this end, we first analyze the phase noise of the detected tone, which is shown in Fig. 6 for both configurations. All comparisons are performed by operating the device in the same lasing regime (first lasing state) and under the same incident optical power at the chip coupler. Owing to the strong thermo-optic effect in silicon OMCCs, the effective detuning is defined relative to the self-consistent lasing condition rather than to a cold-cavity resonance. This ensures that the phase-noise measurements shown in Fig. 6 correspond to identical operating conditions for both coupling schemes. It can be seen that the phase noise curves display the characteristic slopes typically observed in self-sustained optomechanical oscillators^18^. Specifically, a $1/f^3$ dependence, attributed to flicker noise, is observed at low offset frequencies; a $1/f^2$ dependence, associated with thermomechanical noise^23^, appears at intermediate offsets; and a flat $1/f^0$ region, corresponding to white phase noise, dominates at large offsets. These features are consistent with the general description given by Leeson’s model^24,25^, confirming the expected noise behavior in free-running PMOs. Figures 6(a) and (b) show the measured phase noise together with fitted slopes for both coupling configurations.

**Fig. 7:**
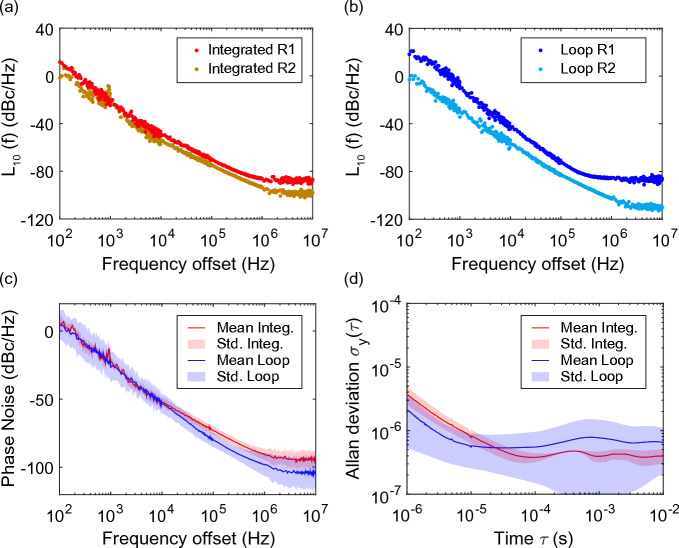
Comparison between loop- and waveguide-coupled setups. (**a**) Phase noise of two independent runs (R1, R2) in the integrated waveguide configuration. (**b**) Same for the loop configuration. (**c**) Mean and standard deviation of the phase noise for both setups. (**d**) Mean and standard deviation of the Allan deviation, showing the superior stability of the integrated waveguide-coupled setup.

The measurements were taken at the respective mechanical frequencies measured at each setup, which may differ slightly due to optomechanical backaction, as explained above. Both were driven to the phonon lasing regime under the same detuning and input power conditions. For a fair comparison, and considering that the frequencies are slightly different, we scaled the results to a common carrier frequency of 10 GHz^3^ using the standard relation:

1 $$\begin{aligned} \textrm{PN}_1 = \textrm{PN}_0 + 20 \cdot \log \left( \frac{f_1}{f_0}\right) \end{aligned}$$

The scaled spectra are shown in Fig. 7. Figure 7(a) and (b) present two independent measurement rounds (R1 and R2) performed on the same device for the integrated waveguide and loop configurations, respectively. Each measurement round was performed after physically decoupling and re-coupling the device, followed by operation in the same phonon lasing state. The two rounds shown correspond to representative extreme cases (lowest and highest phase noise) and are intended to illustrate the range of variability observed under independent coupling conditions. This approach allows us to assess the reproducibility of independent measurements and to evaluate how coupling-related variability is reflected in the uncertainty of the extracted phase-noise spectra. In the loop configuration, re-coupling may involve slight variations in the position of the tapered loop relative to the cavity, while in the edge-coupled configuration it can lead to small changes in the fiber-to-waveguide coupling ratio. While in R2 of the loop setup, a slightly lower phase-noise floor is observed at large frequency offsets, the integrated waveguide-coupled configuration exhibits higher reproducibility across both runs. This indicates that the edge setup provides more consistent and reliable performance, as it may be expected since slight fluctuations of the coupling element (the fiber loop) are removed, as noted in Fig. 1(c). To quantify this, Fig. 7(c) compares the mean and standard deviation of the phase-noise spectra for both configurations. The shaded regions clearly reveal a larger variability in the loop setup, which can be attributed to the way the tapered fiber loop is placed in contact with the cavity for evanescent coupling, making the configuration more sensitive to environmental fluctuations, such as mechanical vibrations and air flow, even though the sample is partially shielded with methacrylate panels. Figure 7(d) shows the same analysis for the Allan deviation, where the discrepancy between both setups is even more pronounced. These results highlight the improved stability and repeatability offered by the mechanically robust waveguide-coupling configuration.

To further evaluate the oscillator stability, we computed the root-mean-square (RMS) timing jitter, a parameter that quantifies signal fluctuations in the time domain, for both coupling configurations and for the two independent measurement rounds (R1 and R2). The RMS jitter can be obtained by integrating the phase noise spectrum over a given frequency range as

2 $$\begin{aligned} J_{RMS} = \frac{1}{2 \pi \nu _0} \sqrt{2 \int _{f_1}^{f_2} 10^{\mathscr {L}(f)/10} df}, \end{aligned}$$

where $f_1$ and $f_2$ are the integration limits. The results are summarized in Table 1, together with representative values reported for state-of-the-art optomechanical oscillators. The jitter values are integrated over different frequency offset ranges, allowing a fair comparison across devices.

For the waveguide-coupling configuration, the two measurement rounds yield values that are consistent within the same order of magnitude across all frequency bands, indicating good repeatability. In contrast, the loop configuration exhibits a significant discrepancy between R1 and R2, with R1 presenting values that are more than an order of magnitude higher than R2. This confirms once again the larger sensitivity of the fiber-loop coupling scheme to environmental perturbations and mechanical drifts.

When compared with previously reported optomechanical oscillators, our edge-coupled device achieves jitter values that are within the same range as those demonstrated in earlier works (see, e.g^26^., and^18^). However, no significant reduction in jitter is observed with respect to the best reported values (e.g^27^., with *∼*10 ps over a broad offset range). This suggests that while the waveguide coupling improves stability relative to the fiber loop, the overall noise performance of our device remains comparable to existing optomechanical oscillators rather than setting a new benchmark. Future improvements will likely require optimization of the cavity design and noise suppression strategies beyond the coupling configuration.

**Table 1:** Jitter comparison across different frequency ranges and OM cavities.

$J_{RMS}$ (ps)	100 Hz – 1 kHz	1 kHz – 10 kHz	10 kHz – 100 kHz	100 Hz – 1 MHz
^26^	-	210.5	36.7	-
^27^	-	-	-	10.1
^9^	92.3	5.3	0.6	92.4
Loop R1	2032.4	153.7	12.2	2038.2
Loop R2	175.2	18.2	2.6	176.2
Edge R1	303.7	33.6	4.0	305.6
Edge R2	567.9	44.0	7.4	569.6

## Conclusions

We have experimentally compared two coupling configurations –fiber-loop and integrated waveguide–in a silicon optomechanical crystal cavity (OMCC) operating in the phonon lasing regime around a mech frequency of 4 GHz. Both configurations enable efficient optomechanical transduction and self-sustained oscillation, but differences arise in their stability performance. Our measurements show that mechanical stability in the fiber-loop configuration is more susceptible to environmental perturbations and vibrations, resulting in a slightly worse performance in terms of phase noise and frequency stability of the generated microwave tone. In contrast, the waveguide-coupled configuration, where light is injected through a lensed fiber into an integrated bus waveguide, exhibits improved reproducibility and reduced drift in both phase noise and Allan deviation measurements. Furthermore, this configuration not only provides enhanced robustness against environmental perturbations, but is also inherently more suitable for integration and packaging, being compatible with standard commercial approaches and thus more practical for real-world applications. Quantitative analysis of the RMS jitter confirms this trend: while both configurations yield values within the same order of magnitude as state-of-the-art optomechanical oscillators, the waveguide-coupled setup provides enhanced temporal stability and lower variability between measurement runs.

## Data Availability

Requests for materials or data should be addressed to Laura Mercadé.
